# The multilayered identity of B cell memory

**DOI:** 10.1038/s41423-025-01377-5

**Published:** 2026-01-06

**Authors:** Vassilis Glaros, Nimmy Francis, Taras Kreslavsky

**Affiliations:** 1 Division of Immunology and Respiratory Medicine, Department of Medicine Solna, Karolinska Institutet, Karolinska University Hospital, Stockholm, Sweden; 2 Center for Molecular Medicine, Karolinska University Hospital, Stockholm, Sweden; 3 Department of Pathology, University of Chicago, Chicago, IL USA

**Keywords:** B cell memory, Humoral immune response, Tissue-resident memory B cells, Immunological memory, B cells

## Abstract

The distinctive feature of the adaptive immune system is its ability to generate immunological memory that can provide defense against subsequent infections. In the case of antibody-mediated immune responses, this memory comes in two cellular forms: plasma cells (PCs) and memory B cells (MBCs). PCs protect against reinfection by constitutively producing antibodies. The presence of a diverse pool of MBCs, which can expand and differentiate into PCs in secondary immune responses, is thought to be particularly important for defense against new pathogen variants. Recent studies have shown that the MBC compartment is far more heterogeneous than previously anticipated. This heterogeneity, among other factors, is shaped by their developmental pathway (germinal center (GC) vs non-GC-derived MBCs), the duration and strength of antigenic stimulation, anatomical and microanatomical localization, and the timing of generation in ontogeny. Combinations of these “layers” of MBC identities can define MBCs’ properties and their fate in recall responses. Here, we review the mechanisms underlying MBC differentiation, maintenance, and reactivation and explore how the layered identity of MBCs contributes to the functions of these cells.

## Introduction

Humoral immune responses to vaccination or infection involve the activation of rare antigen-specific B cells, their clonal expansion and differentiation into antibody-secreting plasma cells (PCs). PCs contribute to immune defense by secreting antibodies that help neutralize and eliminate invading agents. While many of these PCs are short-lived, some are maintained for years or even decades as long-lived plasma cells (LLPCs) that continue to secrete antibodies, serving as a first line of defense upon pathogen reencounter.

Some activated B cells, instead of undergoing terminal differentiation to the PC lineage, exit the cell cycle and persist in a quiescent state as memory B cells (MBCs). Upon subsequent antigen exposure—either in secondary immune responses or after rerecruitment into the primary immune response if the antigen persists—MBCs reinitiate the cascade of B cell activation and differentiation. This expanded pool of antigen-specific B cells—greater in number and often functionally distinct from the naïve precursors present before initial exposure—ensures a faster and more robust response if the pathogen manages to bypass the existing antibody-mediated barrier. Notably, recent evidence suggests that the MBC compartment is more clonally diverse than the PC compartment is [1–4] and may play a crucial role in mounting effective responses against novel pathogen variants [3–7]. As the maintenance of LLPCs, cells constantly secreting large amounts of antibodies, is energetically costly, archiving a broader repertoire of antigen-specific B cells within the quiescent MBC compartment may represent a more economical strategy for preserving the diversity of antigen-specific B lineage cells over time.

Historically, studies of B cell memory have focused on class-switched B cells (cells that have undergone switching from IgM/IgD to other antibody isotypes during the course of the immune response), which express a few canonical memory markers and recirculate between secondary lymphoid organs. While these studies have provided valuable insights, they represent a somewhat limited view of the true diversity within the MBC pool. It is now evident that the MBC compartment is highly heterogeneous and includes sizable populations of unswitched cells, which are sometimes phenotypically indistinguishable from naïve B cells in terms of the currently used cell-surface markers. Moreover, it becomes increasingly clear that nonrecirculating tissue-resident MBCs can be found in some anatomical locations where they ensure rapid local recall responses. Finally, MBCs can be heterogeneous in terms of their developmental origin (e.g., germinal center (GC) vs non-GC-derived MBCs), the duration and strength of antigenic stimulation they have experienced, and the timing of their generation during ontogeny. These “layers” of MBC identity, discussed in detail in this review, in turn determine the functional properties of MBCs in secondary immune responses.

## GC-dependent and independent MBCs

Antigen-dependent B cell differentiation is an unusual developmental process in which both main cellular outputs—PCs and MBCs—can arise through two distinct routes, either directly from early activated B cells or after participation in the GC reaction [8]. To elucidate the mechanisms that regulate the generation of MBCs via these pathways, in the following sections, we discuss how the developmental trajectories of MBCs diverge from those leading to alternative fates. We begin with the better-characterized fate decisions within GCs (“Memory formation in GCs”) and then turn to the less understood events in early B cell activation that lead to the generation of GC-independent MBCs (“Pre-GC memory formation”). Finally, we compare the resulting MBC populations, highlighting both shared and distinguishing features and considering how their developmental origins influence their behavior upon reactivation (“Comparison of _GC_MBCs and _E_MBCs”).

### Memory formation in GCs

#### The GC reaction

**Fig. 1:**
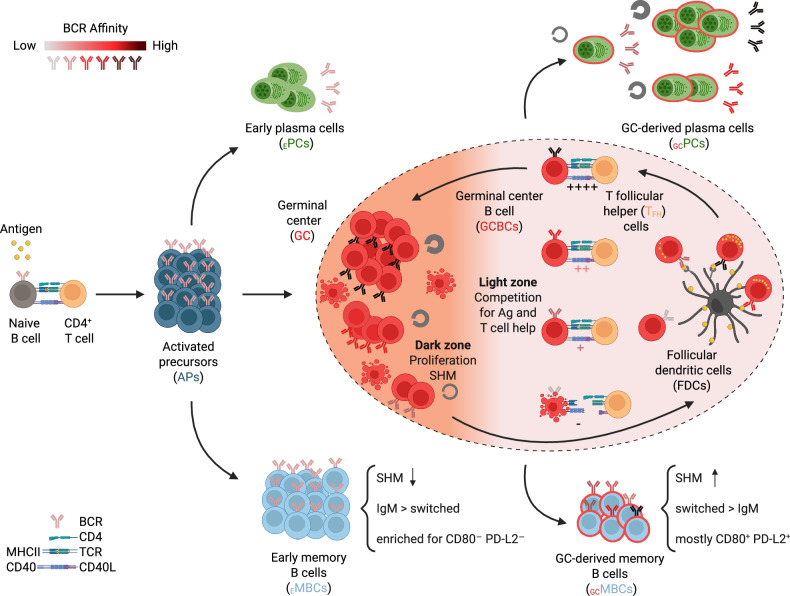
Cell fate decisions upon B cell activation. Upon activation, naïve B cells give rise to tripotent activated precursors (APs). APs can give rise to early plasma cells (_E_PCs) or early memory B cells (_E_MBCs) or differentiate into germinal center B cells (GCBCs) by initiating a specialized effector program enabling their participation in the germinal center (GC) reaction. In the GC reaction, through iterative rounds of competition for antigen and T cell help, proliferation and mutation of their antibody genes, GCBCs can improve their affinity for the antigen. Some GCBCs differentiate into GC-derived PCs (_GC_PCs) or MBCs (_GC_MBCs). See the text for further details

B cells exhibit the remarkable capacity to refine the binding strength of their B cell receptors (BCRs) to antigens—a process known as affinity maturation. This evolution of antibody affinity occurs within transient microanatomical structures called germinal centers (GCs), which form in the B cell follicles of secondary lymphoid organs [9–12] as well as in tertiary lymphoid structures (TLSs) [13]. B cells participating in the GC reaction ultimately give rise to GC-derived plasma cells (_GC_PCs) and GC-derived memory B cells (_GC_MBCs) (Fig. 1). Although most GCs emerge temporarily in response to infections or immunizations, some lymphoid organs, such as Peyer’s patches (PPs) in the gut, can host persistent GCs [14, 15]. The GC reaction has been predominantly studied in the context of T-dependent responses; however, it is important to note that short-lived GCs can also form in response to T-independent antigens [16–18]. These GCs can support the generation of _GC_MBCs and _GC_PCs [18], although the mechanisms and functional significance of such GC reactions remain to be further elucidated.

GCs are formed in B cell follicles by antigen-specific B cells that, after encountering their cognate antigen and receiving T cell help at the T-B border, undergo a burst of proliferation [19] (Fig. 1). Early GCs are polyclonal, often initiated by multiple “founder” clones [20], and remain accessible to additional antigen-specific B cells that can enter the GC reaction later on and join the ongoing response [21–25]. GCs are architecturally organized into two functionally distinct zones [26–28]: the dark zone (DZ), where B cells proliferate and introduce mutations into their immunoglobulin (Ig) genes through somatic hypermutation (SHM) [29], and the light zone (LZ), where mutated B cells with varying antigen affinities migrate to and undergo a two-step selection process [27, 28, 30–33] (Fig. 1). First, they compete for antigen displayed by follicular dendritic cells (FDCs) [34–36]. Cells that successfully acquire antigen present its epitopes on MHC class II molecules to GC T follicular helper (GC-T_FH_) cells—a specialized subset of CD4⁺ T cells that reside within GCs [37–39]. These T_FH_ cells, in turn, provide GC B cells (GCBCs) with prosurvival and mitogenic signals through the CD40-CD40L interaction and cytokine secretion, thereby promoting positive selection [28, 40–42]. Positively selected GCBCs then continue their diversification within the GC. The GC reaction is an iterative process that can continue for weeks or even months [43–46], during which surviving B cells undergo many rounds of proliferation, SHM and selection [27, 28, 30–33]. Throughout this process, GCBCs continuously choose between four distinct fates: cell death [33], continued participation in the GC reaction [27, 28, 30–33], and differentiation into _GC_PCs or _GC_MBCs [47, 48]. In the following two sections, we discuss how the signals received by GCBCs are translated into these cell fate decisions.

#### Cell fate decisions in GCs

Most GCBCs are thought to undergo apoptosis during the GC reaction, either because of a lack of positive selection signals in the LZ or because of SHM in the DZ that generates nonfunctional BCRs [33]. While it was initially thought that GCBCs with lower-affinity BCRs would be predominantly eliminated by apoptosis due to the lack of survival signals coming from BCR signaling and/or T cell help [12], direct evidence for this is lacking. In fact, comparisons of the ratios of lower-affinity B cells to higher-affinity B cells within the apoptotic and nonapoptotic compartments of the LZ revealed no significant differences [11, 33]. It is therefore conceivable that reduced proliferation rather than increased apoptosis is the main driver of the elimination of lower-affinity clones over time. Indeed, higher-affinity GCBCs capture and present antigen to T_FH_ cells in the LZ more efficiently [28, 49, 50] (Fig. 1), resulting in stronger delivery of T cell-derived signals, increased mTORC1 signaling and greater upregulation of the transcription factor MYC, which in turn leads to a greater burst of proliferation upon their reentry into the DZ [51–53]. Thus, even if higher- and lower-affinity clones experienced similar apoptosis rates, higher-affinity B cells are more likely to undergo additional rounds of proliferation and SHM in the DZ, increasing their representation in subsequent cycles of selection [27, 28, 30–33].

It is important to note that the GC reaction is an inherently “noisy” process, and GC dynamics are not governed solely by affinity-based interclonal competition in a strictly deterministic fashion. Indeed, a recent analysis of hundreds of GC reactions from monoclonal B cells specific for a model antigen revealed that, while an organism-level increase in affinity was reliably achieved, individual GCs exhibited broad variation in both the efficiency and trajectories of affinity maturation [54], highlighting the stochasticity of selection events in GCs. It is conceivable that this “noise” in the selection events could be introduced by constraints in access to antigen and T cell help, as well as by “jackpot” events such as acquisition of antigen aggregates or even antigen-loaded fragments of FDCs. Thus, while relative BCR affinity is a major determinant of cell fate decisions in GCs on average, the fate of an individual GCBC at a given timepoint may be heavily influenced by stochastic factors.

**Fig. 2:**
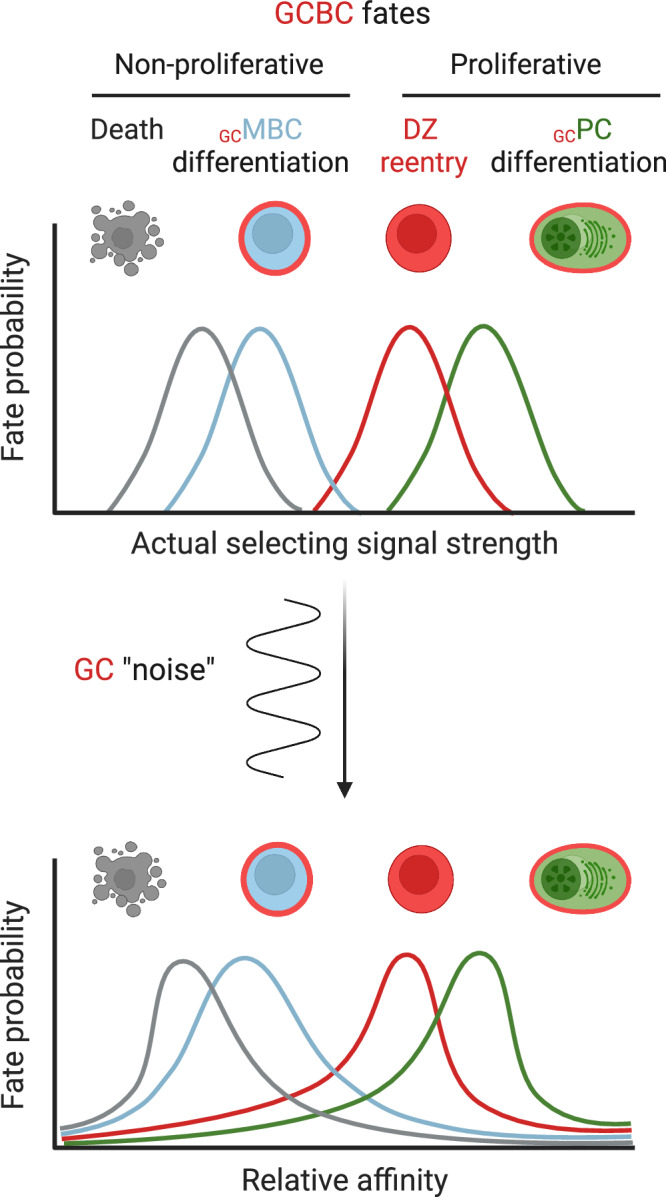
A possible model of cell fate decisions in GCs. The relative affinity of a GCBC’s BCR for an antigen influences the strength and duration of positively selecting signals (signals from BCR and T_FH_ cells) and may instruct cell fate decisions along the axis: death (absence of positively selecting signals) < _GC_MBC differentiation < DZ reentry < _GC_PC differentiation (top). However, the exact boundaries between these outcomes are likely to be strongly blurred by stochastic events in the GC environment (bottom)

Despite this stochasticity, the _GC_PC compartment is enriched for higher-affinity clones compared with both contemporaneous _GC_MBC and GCBC populations [2, 55–57], and BCR transgenic B cells with higher affinity BCRs generate more PCs than B cells bearing a lower affinity BCR recognizing the same antigen [58]. These findings suggested that stronger T cell help (and/or stronger BCR signaling) may preferentially instruct higher-affinity clones to differentiate into _GC_PCs. However, in line with the inherent stochasticity of the GC reaction discussed above, tracing of _GC_PCs in polyclonal responses has demonstrated that even lower-affinity clones can make some contribution to the _GC_PC compartment [59–61]. These results indicate that _GC_PC differentiation is compatible with a spectrum of affinities rather than being restricted to clones at the highest end of the affinity range (Fig. 2). In addition to the selection events taking place within GCs, _GC_PCs remain transiently proliferative after GC egress, with their expansion capacity preprogrammed by the magnitude of T_FH_ help received during LZ selection [56]. This preferential post-GC expansion of higher-affinity _GC_PCs contributes to the enrichment of high-affinity cells within the _GC_PC compartment [56]. How much the formation of the high-affinity _GC_PC compartment depends on GCBC commitment to the PC lineage driven by BCR signaling and T cell help, as opposed to postcommitment expansion of higher-affinity PCs, remains to be fully elucidated.

#### _GC_MBC differentiation

Although several insights have been provided into how affinity maturation progresses in GCs and how the _GC_PC compartment is formed over time, the mechanisms shaping the _GC_MBC pool remain much less well defined. A series of studies have demonstrated that the _GC_MBC compartment is seeded by B cells spanning a broad spectrum of antigen affinities, including both lower- and higher-affinity clones [1, 2, 62, 63], with some MBCs exhibiting extremely low affinity for their antigen [1]. Strikingly, a recent study suggested that the majority of _GC_MBCs, unlike contemporaneous GCBCs, were unable to bind the antigen used for immunization [1]. However, these MBCs were antigen specific, as antibodies cloned from such cells were able to interact with the antigen when the valency of the interaction was increased by multimerization [1]. In addition to their lower average affinity, _GC_MBCs also exhibit greater clonal diversity compared to _GC_PCs [1–4]. This diversity of MBCs, which includes cells of very low affinity, is thought to be beneficial for responses against pathogen variants. Indeed, the importance of this diversity in recall responses has been demonstrated by studies showing that protective antibodies against flavivirus and influenza variants arise primarily from the rapid differentiation of MBCs into PCs rather than from LLPCs established during primary infection [3, 4, 7].

In contrast to DZ reentry or _GC_PC fate commitment, which are linked to bursts of proliferation, _GC_MBC differentiation is associated with a transition to quiescence. Immunization experiments using cell cycle reporter mouse lines have revealed the presence of antigen-experienced, MBC-like cells carrying SHMs in their BCRs among cells with the GCBC surface phenotype and/or among cells physically located in GCs [63, 64]. These cells display features more consistent with MBCs than with typical GCBCs, including quiescent G_0_ cell cycle status, upregulation of cell-surface markers typical for naïve and memory B cells, and reduced expression of the transcription factor BCL6, a master regulator of the GCBC transcriptional program [63, 64]. Multiple studies have identified similar or overlapping populations exhibiting MBC-like characteristics within the GCBC compartment, which have been used to investigate the mechanisms underlying _GC_MBC differentiation [62–66]. Notably, across these studies, these putative _GC_MBC precursors were consistently enriched among GCBCs with LZ-associated surface phenotype [62–66]. These findings, along with evidence from experiments showing enhanced MBC output from CXCR4-deficient GCBCs (which are confined to the LZ) [67], support the notion that the GC LZ may serve as the primary site of _GC_MBC commitment. Overall, attenuation of the GC program in the LZ, coupled with a transition to quiescence, seems to constitute the key process driving _GC_MBC differentiation.

**Fig. 3:**
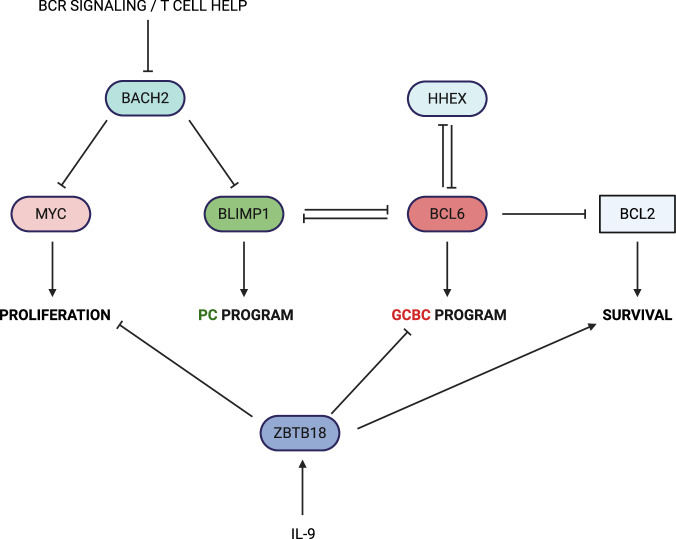
Molecular regulation of _GC_MBC differentiation. _GC_MBC differentiation includes downregulation of the GCBC transcriptional program, transition to quiescence, and upregulation of prosurvival factors. This figure summarizes the role of the factors discussed in the “_GC_MBC differentiation” section in each of these three processes. Lines depict both direct and indirect regulation. See the text for further details

Since the _GC_MBC compartment appears to be enriched for relatively low-affinity clones [1–3, 62] and _GC_MBC commitment is associated with a transition to quiescence rather than a proliferative state, a key question arises: how do these cells avoid apoptosis during this process? Genetic ablation of BCR expression in GCBCs has been shown to trigger apoptosis in candidate _GC_MBC precursors [63], making it unlikely that cells lacking positively selecting pro-survival signals (or, at the very least, lacking tonic BCR signaling [68–70]) in the LZ could successfully develop into _GC_MBCs. This requirement for positive selection signals, together with the enrichment of relatively low-affinity clones in the _GC_MBC compartment and their transition to quiescence within GCs, supports the notion that GCBCs receiving signals strong enough to sustain survival but too weak to trigger proliferation are preferentially directed toward the _GC_MBC fate (Fig. 2). One factor implicated in the “translation” of weak positive signals into _GC_MBC differentiation is the transcription factor BACH2, which is expressed in naïve and activated B cells but not in PCs [2, 71, 72]. LZ GCBCs with lower-affinity BCRs that receive weaker T cell help have been shown to upregulate BACH2 and decrease mTORC1 signaling [63] (the latter is driven by T cell help that promotes positive selection and proliferation within GCs [73]). BACH2 upregulation appears to be directly linked to the transition to quiescence, as BACH2 ablation in GCBCs leads to hyperactivation of mTORC1 and elevated MYC expression [63], both of which promote proliferation in GCs [52, 53, 73, 74]. In parallel, BACH2 has been shown to actively repress the expression of *Prdm1*, encoding the transcriptional regulator BLIMP1, which is essential for commitment to the PC lineage [71, 75] (Fig. 3). Notably, these observations, together with the established role of the BACH2-BLIMP1 axis in T cell differentiation, where BACH2 promotes memory cell fate by antagonizing BLIMP1-driven effector differentiation [76–79], point toward conserved regulatory circuits governing memory versus effector differentiation across the B and T cell branches of the adaptive immune system [76].

The antiapoptotic protein BCL2 may play a central role in supporting the survival of GCBCs differentiating into _GC_MBCs. In contrast to the majority of GCBCs, which express very low levels of BCL2, the candidate _GC_MBC precursors have been shown to upregulate this prosurvival factor [63]. Moreover, studies using *Bcl2*-transgenic mice demonstrated that inhibition of apoptosis within GCs leads to a significant increase in MBC numbers [80, 81]. However, it remains unclear whether this increase was due to enhanced survival of GCBCs in general, increased survival and MBC differentiation of GCBCs that otherwise would undergo apoptosis, increased survival of MBCs after their differentiation, or a combination of these mechanisms [80, 81]. Interestingly, a more recent study showed that constitutive *Bcl2* expression in GCBCs can allow cells with nonfunctional BCRs generated in the DZ to become quiescent and begin expressing MBC-associated markers, although these cells fail to transition from the DZ back to the LZ [82]. While the exact mechanisms that induce *Bcl2* expression remain unclear, downregulation of the GC program may play a key role, as the transcriptional repressor BCL6, a master regulator of the GCBC gene expression program, has been shown to repress *Bcl2* expression in GCBCs [83] (Fig. 3). BCL6 also appears to repress *Hhex* expression, which encodes the transcription factor HHEX [66]. HHEX was suggested to positively regulate _GC_MBC generation through the downregulation of BCL6 and release of BCL6-mediated repression of BCL2 [66, 84] (Fig. 3). Overall, these observations suggest an antagonism between the GCBC program maintained by BCL6 and the prosurvival program necessary for _GC_MBC differentiation.

Recently, the transcriptional repressor ZBTB18, which is induced in _GC_MBC precursors by IL-9 [64], has been implicated in the regulation of all key aspects of _GC_MBC differentiation, including the suppression of cell cycle-associated genes, the repression of some GCBC program components, and the promotion of _GC_MBC precursor survival [85] (Fig. 3). The availability of another T_FH_-derived cytokine, IL-4, has also been shown to influence _GC_MBC differentiation, with one study suggesting that IL-4-mediated downregulation of BCL6 may facilitate GC exit [86]. However, assessing the overall effect of IL-4 on _GC_MBC differentiation has been challenging, as it regulates multiple key processes throughout B cell responses, including GC initiation [86, 87], and has been reported to either promote or suppress _GC_MBC development, depending on the timing of its experimental manipulation [86, 88].

Collectively, these observations suggest that relatively low levels of selection signals may allow certain GCBCs to exit the GC reaction as _GC_MBCs. These low-level signals appear to be translated into downregulation of the GCBC transcriptional program, a transition to quiescence, and upregulation of prosurvival factors such as BCL2 (Fig. 3). It is plausible that this quiescence-based, rather than proliferation-driven, mode of generation contributes to the increased clonal diversity observed within the _GC_MBC compartment [1–4]. Indeed, mechanisms that translate affinity differences into differential proliferation rates may result in strong overrepresentation of certain clones, thereby reducing diversity. In contrast, the differentiation of _GC_MBCs is not associated with such clonal outgrowth and therefore may preserve greater clonal diversity. In addition, as the _GC_MBC fate is compatible with a broad range of affinities—and as affinity-reducing and neutral mutations should be more common than those that increase affinity—this may further promote diversification of the _GC_MBC repertoire.

Putting the full spectrum of cell fate outcomes in the GC reaction into perspective, the insights discussed above suggest that the four possible fates of GCBCs can be broadly classified into two major trajectories. Stronger positively selecting signals drive proliferative outcomes, including DZ reentry for continued participation in the GC reaction, as well as differentiation into _GC_PCs. In contrast, weaker—or even absent—positively selecting signals can lead to either cell death or entry into a quiescent state, marking commitment to the _GC_MBC fate. While the strength of positive selection signals clearly influences the choice between “proliferative” and “quiescent” trajectories, the hierarchy of cell fate decisions within these categories remains less well defined. It is conceivable that all four fates can be positioned along one axis of positively selecting signal strength (e.g., cell death < _GC_MBC differentiation < DZ reentry < _GC_PC differentiation) (Fig. 2). If so, the exact boundaries between these outcomes are likely to be strongly blurred by stochastic events in the GC environment [54] (Fig. 2). For example, a high-affinity B cell that fails to engage with a T_FH_ cell by chance may enter the _GC_MBC pool, explaining the existence of high-affinity MBCs. Conversely, a lower-affinity cell that happens to capture a large antigen aggregate might expand disproportionately within the GC and/or _GC_PC compartment. Finally, it is also conceivable, although perhaps less likely, that fate decisions within each branch—proliferative or quiescent—are entirely stochastic. Overall, the findings described above illustrate the complexity and dynamic nature of cellular decision-making within the GC. As discussed below, conceptually similar lineage choices also take place at the earlier stages of B cell activation prior to initiation of the GC reaction.

### Pre-GC memory formation

#### Early B cell activation

Although the GC pathway is the most extensively studied route for MBC and PC generation, both cell types can also arise directly from early activated B cells (or from reactivated MBCs) independently of the GC reaction (Fig. 1). After recognizing their cognate antigen in the follicles of secondary lymphoid organs, B cells internalize it, process it for presentation on MHC class II molecules [89, 90], migrate to the outer edge of the follicle—the border between the B and T cell zones—and engage with antigen-specific CD4⁺ T cells [89, 90]. Productive interactions between MHC II-peptide complexes on B cells and T cell receptors on CD4⁺ T cells trigger the delivery of T cell help, including costimulatory prosurvival and mitogenic signals through CD40-CD40L interactions and the secretion of cytokines such as IL-4 and IL-21, which together drive the rapid proliferation of B cells at the follicular perimeter [19, 89–91]. These activated B cells acquire a unique cell-surface phenotype characterized by the coexpression of markers associated with both naïve/memory (CD38, CCR6) and activated/GCBC (CD95, GL7) cell states [92–95]. Like naïve B cells, these cells can initially retain IgD expression [92–95], although some of them undergo class-switch recombination (CSR) early on and therefore lose IgD and IgM BCRs [92, 96, 97]. These cells express intermediate levels of the transcription factor IRF4 [95, 98, 99], which is required for both PC and GCBC differentiation [99, 100], and exhibit either absent or low expression of BCL6 [95, 98, 99, 101], the key transcriptional regulator of GCBC fate [102, 103]. This population, often referred to as “activated precursors”, is thought to represent common tripotent progenitors of GC-independent early plasma cells (_E_PCs), early memory B cells (_E_MBCs) and GCBCs (Fig. 1). In support of this tripotency, limiting dilution cell transfer experiments, which enable tracking of the fate of single naïve B cells in vivo, have demonstrated that an individual naïve B cell is capable of generating all three lineages—GCBCs, _E_PCs, and _E_MBCs—although cell death may prevent some clones from contributing to all compartments [104]. These lineage choices take place in the first days after antigen encounter, and at least in some experimental systems, the split between the three lineages is evident by day 4 after immunization [92, 95, 98, 99, 105]. Although these pre-GC events are much less well characterized than those occurring within GCs are, it is evident that, like the lineage choices made in GCs, these fate decisions are largely shaped by the availability of antigen and T cell help.

#### Cell fate decisions early upon B cell activation: _E_PC and GCBC differentiation

As discussed in the section “Cell fate decisions in GCs”, increased access to antigen and/or T cell help in GCs favors _GC_PC generation. Similarly, injection of antigen or anti-CD40 antibody during the early stages of the response has shown that increased availability of antigen and T cell-derived signals shifts the composition of the early activated B cell compartment toward the _E_PC lineage [92, 98]. Under physiological conditions, where antigen can become limiting very early on [106–108], resulting in increased competition, BCR affinity likely plays an important role in determining access to antigen and T cell help. Indeed, experiments comparing the fate of early activated BCR transgenic B cells in response to immunogens of varying affinities have shown that higher-affinity interactions increase the frequency of _E_PCs relative to that of GCBCs or _E_MBCs [109–111]. At the single-cell level, while the limiting dilution cell transfer experiments described above clearly demonstrated that individual naïve B cells can generate all three lineages, their fate varied with antigen affinity. While in response to both higher-affinity antigens and lower-affinity antigens, naïve B cells showed similar potential to generate GCBCs [104], higher-affinity immunization favored _E_PC formation, whereas _E_MBCs were generated more frequently in response to lower-affinity antigen [104]. Overall, these observations suggest that increased antigen availability, the expression of higher-affinity BCRs, and stronger T cell-derived signals favor the generation of _E_PCs.

The experiments described above indicate that GCBCs can, in principle, be generated by both higher-affinity and lower-affinity B cells; however, in a competitive setting, lower-affinity clones compete poorly for T cell help and may not contribute efficiently to the GCBC compartment [94, 112–115]. Although the precise quantity or quality of signals that might favor GCBC over PC differentiation remains unclear, it is conceivable that GCBC differentiation is induced by intermediate levels of BCR signaling and/or T cell help. However, unlike PC differentiation, which can be easily induced in vitro by anti-IgM or anti-CD40 stimulation in the presence of cytokines, the GCBC fate, to our knowledge, cannot be induced by a simple reduction in the levels of these stimuli, suggesting that GCBC specification requires a more complex regulatory framework. In line with this notion, an interesting alternative hypothesis suggests that interruptions in CD40-CD40L signaling, rather than signaling strength per se, may bias differentiation toward the GCBC lineage [98]. Indeed, short-term removal of the anti-CD40 antibody after in vitro B cell stimulation was sufficient to induce BCL6 upregulation in a fraction of cultured B cells [98], and the transient decrease in T cell-derived signals in vivo through anti-CD4 or anti-CD40L treatment during the early stages of B cell activation seems to increase the propensity of activated B cells to generate GCBCs [98]. In contrast, increasing and/or prolonging signals associated with T cell help by injecting anti-CD40 antibodies blocked GCBC generation and induced a wave of _E_PCs [98].

Finally, it is also important to note that, at least in some immunization scenarios, _E_PCs and GCBCs emerge asynchronously, and _E_PC generation is restricted to a narrow time window very early in the response before the emergence of the first GCBCs [92]. This suggests that at a given timepoint, an activated precursor may choose between turning on a single possible “effector program” (either _E_PC or GCBC) or remaining in the precursor state rather than making an actual choice between the induction of the GCBC and _E_PC programs. A very large fraction of these activated precursors, however, do not induce either the GCBC or _E_PC molecular programs. Instead, as discussed in the next section, they undergo “differentiation by default” to the _E_MBC lineage [92].

#### _E_MBC differentiation

While most studies of B cell memory have focused on _GC_MBCs, it has long been recognized that MBCs can also arise from cells that never participate in the GC reaction. Early evidence for such generation of MBCs outside of GCs comes from studies in mice lacking BCL6 in their hematopoietic compartment [116], as well as from patients with CD40L deficiency [117], both of which are unable to form functional GCs but still generate detectable populations of MBCs. Further support for a GC-independent MBC pathway came from studies that showed the early emergence of antigen specific, largely unswitched cells with an MBC phenotype before the appearance of the first GCBCs [93, 118]. Moreover, at least in some immunization scenarios, most early activated B cells start exiting the cell cycle within the first few days of the response, giving rise to a large wave of such _E_MBCs rather than contributing to the GCBC or _E_PC compartments [92]. Notably, these _E_MBCs are also not affected by BCL6 deficiency [93]. Finally, BrdU pulse-chase experiments that allow tracking of B cells exiting the cell cycle in the first days of the response, prior to GC formation, demonstrated that _E_MBCs can be long-lived [119].

Reminiscent of the MBC differentiation in GCs, which is driven by weaker—or even absent—access to antigen and/or T cell help, the transition to quiescence, which marks the generation of _E_MBCs during early B cell activation, also appears to be linked to limited or missing signals that would otherwise drive sustained proliferation and differentiation into GCBCs or _E_PCs. In line with that notion, it was shown that the early cell cycle exit of the majority of activated B cells that marks _E_MBC differentation is driven by a rapid decline in antigen abundance outside of GCs during the first few days of the response [92]. Indeed, in common immunization scenarios, antigen is readily detectable across B cell follicles in the first hours after immunization, but its presence becomes rapidly restricted to GCs thereafter [106–108]. The provision of additional antigen several days after immunization prevents the transition to quiescence associated with _E_MBC differentiation and instead promotes the differentiation of early activated B cells to the _E_PC lineage [92]. A similar dominance of quiescent _E_MBCs early in the response that coincided with rapid loss of antigen outside of GCs has been observed after immunization in nonhuman primates, suggesting that the pathway leading to _E_MBC differentiation is evolutionarily conserved [92].

Thus, although the _E_MBC fate appears to be adopted by a large fraction—if not the majority—of activated B cells from very early stages upon activation [92], this state seems to reflect not an active differentiation process but rather a passive transition to quiescence driven by the loss of activating signals. This transition is marked by minimal transcriptional changes, in sharp contrast to the rapid and extensive transcriptomic divergence observed in cells committing to either the GCBC or _E_PC fates [92]. These findings suggest that _E_MBC differentiation may represent a “default” pathway in which cells largely retain the transcriptional identity of the common precursor state. Moreover, these findings could imply that the relative abundance of _E_MBCs within the MBC pool may be shaped primarily by the extent of initial expansion of early activated B cells in the first days of the response, which in turn can be influenced by the naïve precursor frequency, affinity of their BCRs to antigen, and antigen abundance early in the response. In support of this idea, it has been shown that mice with a greater frequency of naïve B cell precursors bearing germline-encoded, high-affinity BCRs for phycoerythrin (PE) developed MBC compartments dominated by IgM⁺ MBCs [120]—a subset likely enriched for _E_MBCs (see the “Shared and distinct properties of _GC_MBCs and _E_MBCs” section below).

Another layer of regulation of _E_MBC abundance may involve competition for extrinsic survival factors in the microenvironment, which could promote the long-term persistence of these cells. The cytokine BAFF, for example, has been suggested to be essential for _E_MBC generation [121] and for the maintenance of both _E_MBCs and _GC_MBCs [121, 122]. Additionally, a recent study suggested that Notch2 deficiency preferentially impairs _E_MBC development [123]. BAFF is essential for the survival of all naïve B cells [124–126], whereas Notch signaling is critical for the development of marginal zone (MZ) B cells [127, 128], which make up a substantial fraction of the naïve B cell pool in the spleen. The reliance of _E_MBCs—and in some instances _GC_MBCs—on maintenance factors shared with naïve B cells supports the notion that the MBC lineage choice may not entail the activation of a complex, specialized differentiation program but rather represents a partial reversion to a naïve-like quiescent state. Nevertheless, as discussed in the following sections, MBCs acquire functional properties distinct from those of naïve B cells, which shape their fate upon reactivation.

### Comparison of _GC_MBCs and _E_MBCs

#### Shared and distinct properties of _GC_MBCs and _E_MBCs

Direct comparison of _E_MBCs and _GC_MBCs has remained challenging until recently, as the utilization of cell-surface markers and the Ig class switching status of the cells does not allow definitive discrimination between the two subsets. In recent years, the development of GC-specific fate-mapping systems using tamoxifen-inducible Cre recombinase driven by regulatory elements of GC-specific genes such as *S1pr2* and *Gcsam* (*Gcet*) has enabled irreversible labeling of GC-derived populations [2, 129]. Such fate mapping allows direct discrimination between _E_MBCs and _GC_MBC, independent of isotype, surface marker expression, or the degree of SHM, which previously served as indirect indicators of GC or non-GC origin. These systems have now enabled phenotypic and molecular comparisons of _E_MBCs and _GC_MBCs as well as assessments of their contributions to the overall memory cell pool. Unexpectedly, such fate-mapping experiments demonstrated that _E_MBCs, which are often considered a minor memory subset, outnumber their GC-derived counterparts across multiple immunization scenarios ([92] and unpublished results). These studies also revealed that cell-surface markers, Ig class switching status, and SHM levels previously used to classify MBC subsets, while informative, are insufficient to definitively distinguish between _E_MBCs and _GC_MBCs [92, 93, 118, 119, 129–132]. Nevertheless, since much of our current understanding of _E_MBC and _GC_MBC biology is based on experiments employing these surrogate markers, we discuss them below.

The Ig class-switching status of MBCs is frequently used as indirect evidence of a history of participation in the GC reaction (or lack thereof). This was initially based on the assumption that CSR occurs predominantly in the GCs. Although recent studies have suggested that CSR may in fact take place primarily prior to GC formation, switched cells gain a competitive advantage in GCs and are therefore enriched in the course of the GC reaction [96, 97, 133]. In line with this notion, multiple studies have shown that MBCs generated early in the response are enriched for unswitched IgM⁺IgD⁻ or IgM⁺IgD⁺ cells, whereas those arising later are predominantly class-switched [92, 93, 118, 119, 132]. However, this distinction is not absolute since class-switched MBCs can be generated at pre-GC stages, albeit as a minority [92, 93, 118, 119]. Moreover, experiments with GC-specific fate mapping have demonstrated that _E_MBCs include a significant fraction of class-switched cells [129], whereas some _GC_MBCs can retain an IgM⁺ [129–131] or even IgM⁺IgD⁺ [132] phenotype. Taken together, these findings indicate that the utilization of class switching status is insufficient to definitively discriminate _E_MBC and _GC_MBC subsets and that even the IgD^+^ compartment can contain some antigen-experienced cells of both GC and non-GC origin.

Studies from the Shlomchik laboratory demonstrated that several subsets of MBCs can be phenotypically distinguished on the basis of the expression of the surface markers CD80, PD-L2, and CD73 [134]. Their subsequent work refined this classification by focusing on CD80 and PD-L2, identifying three subsets: double-positive (DP: CD80⁺PD-L2⁺), single-positive (SP: CD80⁻PD-L2⁺), and double-negative (DN: CD80⁻PD-L2⁻) MBCs [135]. A recent study using GC-specific fate mapping revealed that while the DP population is enriched for _GC_MBCs and that DN MBCs consist almost exclusively of _E_MBCs, this distinction is again not absolute, as a considerable proportion of DP cells appear to originate from the non-GC pathway [129].

In addition to the cell-surface phenotype, the level of hypermutation can also provide an indirect record of the GC history of a cell. Indeed, _GC_MBCs display higher mean levels of SHM in their immunoglobulin variable region genes than do their non-GC-derived counterparts [129, 132]. However, the mutation load within each population is highly variable [129, 132], and some _GC_MBCs display SHM levels similar to those of _E_MBCs and vice versa [129, 132].

Taken together, these results indicate that _E_MBCs are enriched for unswitched, DN MBCs with relatively low levels of SHM, whereas _GC_MBCs are more commonly class-switched, DP cells with higher SHM levels (Fig. 1). However, none of these characteristics are fully exclusive to either subset, and the phenotypic properties of the two populations significantly overlap.

#### _GC_MBC vs _E_MBC function upon reactivation

Despite their overlapping phenotypes, _E_MBCs and _GC_MBCs clearly exhibit distinct differentiation potentials upon reactivation. Studies analyzing the fate of IgM⁺ and class-switched MBCs—either upon boost immunization or adoptive transfer of MBCs into unimmunized recipients followed by immunization—have suggested that IgG1⁺ MBCs (enriched for _GC_MBCs) are biased toward PC differentiation, whereas IgM⁺ MBCs (enriched for _E_MBCs) are more likely to reenter secondary GCs [118, 136]. Similar observations were made using CD80 and PD-L2 expression as MBC subset markers: DP MBCs (enriched for _GC_MBCs) tend to be more prone to PC differentiation, whereas DN MBCs (enriched for _E_MBCs) more efficiently reenter secondary GCs [129, 135]. The propensity of _GC_MBCs to generate PCs rather than enter secondary GC reactions was directly shown by GC-specific fate-mapping experiments, which demonstrated that upon booster immunization, fate-mapped _GC_MBCs are predominantly biased toward PC differentiation and contribute only minimally to secondary GC formation [137, 138]. Instead, non-fate-mapped cells, a compartment that can include both naïve B cells [137, 138] and _E_MBCs, dominated the secondary GCBC pool under these conditions. Finally, a subsequent study revealed that these non-fate-mapped cells found in secondary GCs were derived primarily from naïve B cells [137]. Taken together, these findings suggest that _E_MBCs possess a greater intrinsic capacity to enter the secondary GC reaction than do _GC_MBCs, but their overall contribution to the secondary GCs remains smaller than that of naïve B cells.

**Fig. 4:**
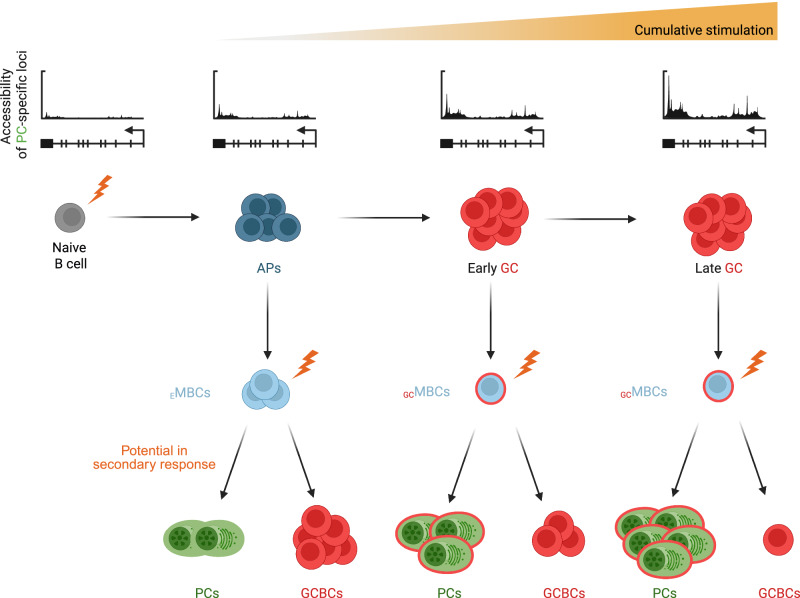
Epigenetic “recording” of the cumulative activation history may preprogram the fate of MBCs in secondary immune responses. Repeated BCR stimulation and/or signals associated with T cell help gradually enhance chromatin accessibility at genes linked to the PC program, such as *Prdm1*, biasing MBCs upon reactivation toward PC differentiation rather than entry into secondary GCs. Such a mechanism could be partly responsible for the differential capacity of _E_MBCs and _GC_MBCs to generate PCs and GCBCs upon reactivation (see section “_GC_MBC vs _E_MBC function upon reactivation” for further details)

Recent transcriptomic analyses suggest that _E_MBCs and _GC_MBCs are closely related at the transcriptional level, with only relatively subtle differences detected between the two subsets ([129, 132] and unpublished observations). The extent to which these differences may explain the functional differences between the two populations remains unclear. Given this limited transcriptional divergence at steady state, it is tempting to speculate that the distinct behaviors of _E_MBCs and _GC_MBCs upon reactivation may be preprogrammed at least in part epigenetically. In line with this notion, it has recently been shown that DN and DP MBCs exhibit distinct epigenetic profiles that may reflect their history of participation—or lack thereof—in the GC reaction, suggesting that these epigenetic signatures may predetermine their responsiveness upon secondary antigen encounter [129]. Mechanistically, this programming may take place at least in part through the epigenetic regulation of BACH2 and BLIMP1 expression [139]. Accumulated stimulation has been shown to progressively increase chromatin accessibility at PC-associated gene loci, including *Prdm1*, thereby increasing baseline BLIMP1 expression and biasing MBCs toward PC differentiation over reentry into the GC response [139] (Fig. 4). These results imply that repeated or prolonged antigen stimulation may gradually shift the MBC potential, contributing to the functional divergence observed between _E_MBCs and _GC_MBCs. In such a scenario, the history of participation in the GC reaction may indirectly determine the reactivation fate of MBCs by increasing the cumulative antigen experience of the cell through repeated cycles of stimulation in the GC. It therefore seems plausible, although remaining to be tested, that a B cell that would undergo repeated or prolonged activation without entering a GC could become biased toward PC differentiation (see also section “Atypical MBCs” below). Conversely, a _GC_MBC generated early in the GC reaction might be less prone to PC differentiation than a _GC_MBC derived from a GCBC that experienced many cycles of stimulation. Taken together, these findings align with an intriguing hypothesis that MBC fate upon reactivation might be programmed not by a binary GC versus non-GC origin but rather by the cumulative history of stimulation that is recorded epigenetically (Fig. 4).

## MBCs in the tissues

Historically, most studies on B cell memory have focused on MBCs that recirculate between secondary lymphoid organs and are therefore capable of responding to antigenic challenges at distal sites, for example, after intramuscular vaccination followed by respiratory infection. In humans, the study of these MBCs is further facilitated by their accessibility from peripheral blood. Recent studies have shown that certain nonlymphoid tissues harbor MBCs, some of which exhibit long-term tissue residency. Moreover, secondary lymphoid organs can also host nonrecirculating MBC populations. In the following sections, we review some of the anatomical sites where MBCs have been identified, examine the evidence supporting their potential tissue residency, and discuss the shared and distinct phenotypic, developmental, and functional characteristics of these cells.

### Lungs and upper respiratory tract

Most of what is currently known about tissue-resident MBCs (BRMs) originates from studies of the lung, a primary site of exposure to respiratory pathogens. The presence of BRMs was first established in influenza-infected mice, where approaches such as parabiosis, intravenous antibody labeling, and pharmacologic blockade of lymphocyte egress via S1PR1 agonists revealed that influenza-specific MBCs could persist within lung tissue and mount rapid responses upon reinfection, strongly supporting their classification as a tissue-resident population [140, 141]. In line with their tissue residency, lung BRMs, unlike their circulating counterparts, exhibit hallmark features of local retention, including the upregulation of CD69 and the concurrent downregulation of S1PR1 [3, 142–146], a receptor that promotes lymphocyte egress from tissues into the circulation [143, 147, 148]. In most of the studies discussed above, lung BRMs have been shown to express the chemokine receptors CCR6 and CXCR3. Although these receptors have been suggested to regulate the migration and retention of BRMs in lung tissue, their exact contributions remain somewhat controversial [142, 149].

Upon respiratory infection, antigen-specific B cells can initiate responses in the mediastinal lymph nodes (medLNs) and within inducible bronchus-associated lymphoid tissue (iBALT)—a TLS that forms locally in the lung parenchyma—highlighting medLNs and iBALT as the most likely sites of BRM origin in the pulmonary environment [140, 145, 149–157]. Additionally, clonal sharing analysis in influenza-infected mice suggested that some BRMs might originate from splenic GCs [150]. Regardless of their site of origin, lung BRMs exhibit transcriptional profiles that are distinct from those of their splenic and lymph node counterparts in both mice and humans [145, 150] but that share some features with the residency program of CD8⁺ tissue-resident memory T cells [158].

While both IgG and IgA antibody responses can be generated following respiratory infection, the BRM pool after primary infection is predominantly composed of IgG⁺ MBCs, with a smaller proportion of IgA⁺ MBCs [140, 151, 152]. Notably, while IgG⁺ MBCs are distributed across both the lung and medLNs, IgA⁺ MBCs are primarily localized within the lung parenchyma, which is in line with the specialized role of IgA in mucosal immunity [149, 151, 156]. This compartmentalization is further supported by the results of experiments showing that systemic immunization with influenza virus fails to induce lung IgA⁺ MBCs, in contrast to intranasal immunization, which effectively promotes their formation [149]. The restricted localization of IgA⁺ MBCs, combined with the spatial association of IgA-secreting PCs with iBALT, suggests that IgA production in the lung is largely driven by these inducible lymphoid structures [145, 149, 151, 156, 157, 159]. Although the presence of IgA⁺ BRMs in the lungs is well established, the mechanisms governing their formation, reactivation, and contribution to protective immunity remain considerably less understood than those of their IgG⁺ BRM counterparts.

While IgG-secreting PC recall responses can originate from both circulating and resident IgG⁺ MBCs, a recent study demonstrated that influenza-boosted mice primed intranasally—a route that favors BRM induction—mounted faster and more localized PC responses than those primed intraperitoneally [156]. In particular, circulating MBCs exhibited delayed differentiation into PCs and were less able to generate PCs in close proximity to infected alveoli, in contrast to their lung-resident IgG⁺ BRM counterparts, which mounted a rapid local response. These IgG⁺ BRMs were not associated with iBALT but were instead dispersed throughout the lung parenchyma, patrolling a much larger area than that occupied by iBALT [154].

Boosting experiments with influenza virus or virus-like particles that lack antigenic similarity to influenza virions have demonstrated that BRMs of diverse specificities—including those unrelated to the invading threat—are rapidly recruited to the site of infection, presumably to “screen“ the local environment for the presence of their cognate antigen [154]. This rapid migratory process is orchestrated by alveolar macrophages, in part through the initiation of a cytokine cascade that involves IFN-γ production by NK cells, which in turn drives the induction of CXCL9 and CXCL10, thereby attracting CXCR3-expressing BRMs to the site of infection [154, 156]. Even more intriguingly, some of these BRMs can differentiate into PCs in response to innate signals alone, producing antibodies despite a lack of specificity for the current threat [156]. This phenomenon suggests that the immune system may, in certain contexts, prioritize speed over precision, particularly in vital tissues such as the lung, where delays in immune responses could be life-threatening. Whether this form of “bystander“ PC differentiation is unique to the lung or also occurs in other tissues remains an open question. It is also unclear whether this capacity is an intrinsic feature of BRMs established during their differentiation in the lung or instead instructed by external cues, potentially specific to highly vulnerable microenvironments such as lung tissue, where rapid local antibody production may be critical for host survival.

Overall, lung BRMs are the best-characterized population of resident MBCs in nonlymphoid tissues, yet their biology remains an area of active investigation, with many questions still unanswered regarding their heterogeneity and functional specialization. For example, the relative contribution and role of GC-derived versus GC-independent MBCs to the lung-resident MBC pool remains unclear. In many of the studies discussed above, BRMs have been identified and visualized via fate-mapping systems driven by the *Aicda* gene, which encodes AID, a key enzyme required for CSR and SHM [142, 154, 156]. However, because both AID expression and CSR can occur outside the GC reaction [92, 96, 97, 160–162], these approaches do not definitively distinguish the developmental origin of BRMs, leaving the precise composition of this population unresolved. Influenza-specific BRMs have frequently been shown to express high levels of CD80, PD-L2, and CD73 [140, 152, 163], which are surface markers associated with _GC_MBCs [129, 135]. However, most studies focusing on BRMs defined by these markers excluded from their analysis IgM⁺ and/or IgM⁺IgD⁺ populations, which are often enriched for _E_MBCs [92–94, 118].

Local immune responses in the respiratory tract are not restricted to the lower airways. Indeed, recent studies have identified antigen-specific GCs and populations of MBCs and PCs within the nasal-associated lymphoid tissue (NALT) of mice following intranasal immunization or infection [164, 165]. In line with these observations, CD69⁺ BRM-like cells have been detected in nasopharyngeal swabs from healthy individuals [166]. Together, these findings suggest that local humoral memory in response to inhaled threats could also be established in the upper respiratory tract, although the candidate BRM compartment at this site requires further characterization.

### Skin

The skin represents another major body surface that is continuously exposed to environmental antigens and microbial challenges, where local immune memory could play an important role. While direct evidence for the presence of MBCs in the skin is lacking, B cells have been identified in the dermis under both homeostatic and inflammatory conditions [167–169], and although they are present in very low numbers, they can include expanded clones [167–169] and class-switched cells [169]. A recent study has shown that exposure to *Staphylococcus epidermidis* induces the formation of dermal TLSs and triggers antigen-specific antibody responses, including the generation of LPPCs that persist for months after exposure [170]. Within these TLSs, skin-associated B cells were shown to undergo class switching to produce IgG2b and IgG2c antibodies, supporting localized humoral responses independent of secondary lymphoid organs [170]. However, whether this cutaneous B cell response can also generate skin-resident B cell memory remains to be tested.

### Gut

Like the respiratory tract and the skin, the gastrointestinal tract represents a highly immunologically active site owing to its constant exposure to dietary antigens, commensal microbes, and potential pathogens. PCs in the gut, which predominantly secrete IgA, arise through both T-dependent and T-independent mechanisms [171–174] and not only protect against pathogens but also shape the composition of the commensal microbiota [171, 173–178]. Studies using oral immunization have demonstrated rapid and enhanced mucosal antibody responses upon secondary exposure, suggesting the presence of MBC populations in the gut [179–183]. Recently, such a population of antigen-specific IgA⁺ MBCs was detected among adoptively transferred model antigen-specific B cells that were activated via oral immunization with an antigen conjugated to cholera toxin [184]. These MBCs were found in PPs and mesenteric lymph nodes (mesLNs) and could be detected even one year after priming [184]. Notably, a recent study employing a B cell specific, tamoxifen-inducible Cre recombinase to label B cells generated during a defined developmental window demonstrated that many labeled IgA⁺ MBCs in PPs and PCs in the lamina propria (LP) of the gut are clonally related [185]. Moreover, these MBCs likely contributed to rapid LP PC reconstitution upon depletion of the latter cells with bortezomib [185]. Taken together, these results suggest that these cells may be part of a gut-resident MBC compartment that contributes to the long-term replenishment of the PC pool in the intestinal LP.

However, whether the intestinal MBC compartment contains bona fide BRM cells remains to be formally tested. It also remains to be determined whether these cells are strictly confined to gut-associated lymphoid tissues (i.e., PPs and isolated lymphoid follicles) or whether they can also populate the LP. Serving as indirect evidence for the latter, in human intestinal samples, excluding epithelial tissues and PPs, MBC-like cells have been identified, with nearly half expressing CD69 [186], a marker widely associated with tissue residency in both B and T cells. Moreover, although evidence supports the presence of _GC_MBCs in the gut [184, 187], the existence and potential roles of non-GC-derived MBCs, whether arising through T-dependent or T-independent pathways, remain to be investigated. In addition, the relative contribution of pathogen-specific and microbiota-specific cells to the MBC compartment of the gut as well as their role in maintaining antibody responses remain to be elucidated.

### Liver

Beyond barrier tissues, the liver represents an organ in which local infection may give rise to MBCs with features of tissue residency, with the most compelling evidence coming from *Ehrlichia muris* infection in mice. In this experimental model, somatically hypermutated IgM⁺ T-bet⁺ MBC-like cells can be found in both the liver and the spleen [188]. In the liver, these cells reside within the tissue, as evidenced by their lack of labeling following intravenous antibody injection [188]. These MBCs are likely generated through a GC-independent pathway (as GCs are absent in the spleen in this infection model) and persist after infection clearance, suggesting their potential role in long-term protection against reinfection [188]. Repertoire analysis of spleen and liver MBCs, although predominantly identifying spleen-specific clones, suggested that approximately half of the liver-derived clones were exclusive to the liver [188]. Additionally, RNA-seq analysis revealed that liver but not splenic MBCs expressed *Cd69* [188]. Moreover, in humans, some studies suggest the presence of atypical MBCs (see the “Atypical MBCs” section below) in the livers of chronic hepatitis B patients [189, 190]. Taken together, these findings indicate the presence of potential tissue-resident MBCs in the liver, but further experiments are needed to assess their migratory properties, the mechanisms underlying their generation, and their possible functional role.

### Evidence for lymph node-resident B cell memory

In addition to the presence of MBCs in nonlymphoid tissues, substantial evidence suggests the existence of resident MBCs at strategic locations within secondary lymphoid organs, enabling them to mount rapid recall responses upon antigen re-exposure. Studies using parabiosis, intravenous antibody labeling, and live imaging of MBCs after immunization or infection have demonstrated that a large fraction of antigen-specific MBCs can persist in draining lymph nodes (dLNs) [140, 191]. Although these MBCs display migratory behavior within lymph nodes similar to that of naïve B cells, which frequently scan the subcapsular sinus (SCS) [191], where the lymph first enters and lymph-borne antigens are captured and displayed by SCS macrophages [192, 193], their overall localization is distinct. Unlike naïve B cells, which show no apparent bias between the inner and outer regions of B cell follicles, MBCs in dLNs are strongly biased toward the outer follicular area close to the SCS [191]. Their positioning in this strategic niche, which is enriched for antigen and memory T_FH_ cells [194], ensures rapid recall responses [191]. Although this localization is dependent on signals from SCS macrophages [195], the precise cues that guide it remain to be identified.

Interestingly, antigen-specific MBCs found in distant nondraining lymph nodes (ndLNs) upon immunization do not show a comparable residency pattern, indicating a unique resident subset in dLNs [195]. Consistent with this, transcriptional differences in dLN and ndLN MBCs have been observed, some of which may underpin their distinct localization patterns within LNs [195]. These observations may have implications for vaccination and boosting strategies, as the two MBC populations respond differently upon reactivation, with MBCs in dLNs more efficiently re-entering secondary GCs following boosting than those in ndLNs do [138, 195, 196]. These findings indicate that the site of booster administration, for example, whether booster vaccinations in humans are given in the same arm or the opposite arm as the primary injection, can influence the quality of the resulting immune response.

### MBCs in the marginal zone of the spleen

While MBCs in the SCS of LNs represent a key population poised to mount rapid recall responses to lymph-borne antigens, substantial evidence also supports the presence of a distinct MBC subset in the marginal zone (MZ) of the spleen. Like the SCS in LNs, the MZ is strategically positioned to intercept circulating antigens since it lies at the interface of red and white pulp and is directly exposed to blood circulating through the splenic sinuses [197]. Its direct exposure to circulating blood makes the MZ an ideal location for rapid immune surveillance of bloodborne pathogens and for mounting immediate responses to systemic threats. The MZ is populated by marginal zone B (MZ B) cells, a specialized subset of B-2 cells that are thought to diverge from the conventional follicular B (FO B) lineage during peripheral maturation in the spleen [197, 198]. While the human spleen has a different anatomy than that of mice [197], B cells with an MZ B cell phenotype can be found within B cell follicles near the red–white pulp border, distal from the T cell zone, which is an area with increased accessibility to bloodborne antigens [197, 199, 200]. The development of naïve MZ B cells critically depends on Notch signaling, [127, 128] which imprints a unique transcriptional identity on this population [201–204]. BCR signaling has also been suggested to play a role in the FO vs MZ B cell fate decision; however, the exact mechanism has been a matter of debate [199, 205–208].

Although the MZ B cell compartment in rodents is thought to be mostly composed of naïve innate-like B cells that, upon activation, rapidly generate PCs producing low-affinity IgM antibodies as a first line of defense against bloodborne pathogens [199, 209–211], the situation appears to be different in humans. Indeed, a large fraction of human MZ B cells are considered to represent MBCs, as judged by the expression of the memory marker CD27 [212, 213] and, more importantly, the fact that many of those cells carry BCRs that have undergone SHM [214–217]. This apparent discrepancy between mice and humans might be explained, at least in part, by the use of mice raised under relatively “clean” laboratory conditions with limited microbial exposure. In support of this notion, immunization studies in mice have shown that MBCs with MZ B cell characteristics (as evidenced by cell-surface marker expression, transcriptional profiles, and localization) can emerge following antigenic challenge [123, 142, 218–222]. These observations suggest that the splenic MZ can serve as a niche for MBCs both in mice and in humans. Notably, despite the reported shuttling of B cells between the MZ and B cell follicles [223], parabiosis experiments have shown that naïve MZ B cells represent largely a spleen-resident nonrecirculating population—at least within the tested 3-week time frame—in contrast to FO B cells, which are distributed equally between parabiotic partners [224]. The retention of these cells within the MZ is mediated by several G protein-coupled receptors, including S1PR1 [225], CNR2 [226] and CD97 [227]. Of note, these molecules are expressed at higher levels in MZ B cells than in their FO counterparts, suggesting a specialized tissue residency program. It is intriguing to speculate that MZ-MBCs could likewise induce this residency program, although this remains to be formally demonstrated.

The developmental origin and functions of MZ-MBCs remain largely unknown. It remains unclear whether these cells arise from early activated B cells, through the GC reaction, or via both pathways and whether they preferentially originate from responses initiated by naïve MZ B cells. Similarly, it is not known whether MZ-MBCs can be generated in response to a broad range of immunological challenges in various locations or whether they are primarily induced by antigenic encounters in the spleen. Functionally, it is tempting to speculate that their strategic localization facilitates rapid antibody production upon reencounter with systemic threats; however, this hypothesis remains untested. Pulse-chase labeling experiments have suggested that naïve MZ B cells persist longer than their FO counterparts in the steady state [185, 228, 229]. Given that longevity is a hallmark of immunological memory, it is tempting to speculate that the splenic MZ could serve as an MBC survival niche. Indeed, a recent study demonstrated that long-lived anti-smallpox MBCs with an MZ B cell phenotype and transcriptional features dominate the switched MBC compartment in the spleens of individuals immunized decades earlier with the smallpox vaccine [230], although it remains unclear whether this dominance reflects preferential generation of such MBCs in response to this vaccine or their preferential long-term survival. Taken together, these findings suggest the intriguing – albeit still speculative – possibility that MZ-MBCs represent a specialized, long-lived, strategically positioned BRM subset that has evolved to mediate rapid recall responses to systemic threats.

### Bone marrow

While the BM has long been recognized as a primary niche for antibody-secreting LLPCs [231–234], some evidence suggests that it also serves as a site for MBC residence. In humans, IgM⁺ mature B cells bearing somatically hypermutated BCRs were reported in BM samples from healthy individuals [235]. Moreover, a recent study revealed the accumulation of antigen-specific MBCs in the BM of immunized mice [222]. These cells are detectable more than one year post-immunization [222]. Most strikingly, BM MBCs had a transcriptional signature and BCR repertoire distinct from those of splenic MBCs [222]. The latter result indicates that there is no complete exchange between the MBC compartments of the two organs and suggests that at least some of the MBCs in the BM may represent resident cells. In line with this possibility, MBCs in the BM expressed higher levels of adhesion molecules, such as integrins α4β1 and α6β1, and were found to be in close contact with stromal cells expressing VCAM-1, the ligand for α4β1 [222].

From a teleological perspective, the reasons for the accumulation of MBCs in the BM remain unclear. It is possible that, similar to PCs, they may benefit from survival signals provided by specialized stromal niches in this tissue. However, unlike PCs, which confer protection at a distance through continuous antibody secretion, the functional advantage of MBCs in the BM is less obvious. One possibility is that these cells can still recirculate and/or be mobilized to peripheral lymphoid organs in the course of secondary immune responses. Alternatively, although the extent to which the antigen can reach the BM via the circulation remains unknown, it is tempting to speculate that this site may support the local activation and differentiation of MBCs into PCs, potentially providing a strategic location for the generation of antibody-secreting cells directly within a niche that also supports their long-term maintenance.

## Additional “layers” of MBC identity

While much of our current understanding of B cell memory stems from studies of MBCs generated in adulthood following vaccination or acute infection, recent studies are beginning to shed light on the unique properties of MBCs that arise under less studied conditions. This, for example, includes B cell memory that emerges early in ontogeny as well as atypical MBCs that are generated and persist under the conditions of chronic antigenic stimulation. In the following sections, we highlight recent advances—as well as remaining open questions—in our understanding of these MBC populations.

### Early-life-origin MBCs

During the neonatal period, the naïve immune system is rapidly exposed to a wave of novel antigens, which can drive the development of LLPCs and MBCs. Moreover, neonatal B cells possess a distinct BCR repertoire as they develop in the absence of terminal deoxynucleotidyl transferase (TdT) activity [236], and they have been suggested to preferentially utilize proximal (i.e., located close to D and J segments) V segments in their Ig heavy chain rearrangements [237–239]. A recent study using a B cell specific, tamoxifen-inducible Cre recombinase for pulse-chase labeling demonstrated that B cell clones generated within the first two weeks of life in mice contribute substantially to antigen-experienced B cell compartments across multiple organs in adulthood [185]. This contribution was particularly pronounced in mucosal tissues, likely reflecting exposure to microbial antigens [185]. The majority of IgA⁺ PCs in both the small intestinal LP and BM, as well as a significant proportion of GCBCs in PPs, were derived from these early-life B cell precursors [185]. Notably, a substantial fraction of both IgA⁺ and IgA⁻ MBCs in PPs (possibly _GC_MBCs, as evidenced by their expression of CD73 and PD-L2 [129, 135]) originated from this early-life-origin (ELO) B cell wave; these cells carried SHMs in their B cell receptors and were clonally related to the PCs in the LP [185].

These observations demonstrate that a substantial fraction of the adult MBC compartment throughout life can be composed of MBCs generated early in ontogeny. It remains to be investigated whether the ontogenetic timing of MBC generation can influence the functions of these cells, both in terms of the extent of their contribution to secondary B cell responses and in terms of the quality of these responses. In addition, the developmental pathways underlying ELO MBC formation remain to be defined, including whether these cells are driven primarily by T-dependent or T-independent stimuli and whether they are generated exclusively by the GC pathway or can also arise independently of the GC reaction. Interestingly, in addition to being enriched in mucosal tissues, ELO B cells are enriched among splenic MZ B cells [185]. As the spleen MZ may serve as a hub for MBCs (see the “MBCs in the tissues” section above), it would be interesting to test whether ELO B cells contribute to the pool of MZ-MBCs in the spleen.

### Atypical MBCs

Atypical B cells (ABCs) are a distinct subset of B cells identified across various immune contexts, including chronic infections, vaccination, and autoimmune diseases [240–243]. In humans, ABCs typically lack classical MBC markers such as CD27 and CD21 and often express the transcription factor T-bet along with distinct surface markers, including CD11c, CXCR3, FCRL4, and FCRL5 [243–248]. A functionally and phenotypically related population has been identified in mice as CD11c⁺CD21^lo^ “age-associated” B cells, as these cells expand with age as well as in the context of chronic infection and autoimmunity [242, 249–255]. Although primarily studied in chronic settings, ABCs can also expand during acute infections, both in mice [221] and humans [247, 256–259], as well as upon vaccination in humans [247, 260, 261]. These expanded ABCs then contract over a period of weeks to months in the absence of restimulation [258, 262, 263].

Despite their phenotypic heterogeneity, accumulating evidence points to a conserved transcriptional program. A recent study revealed that ABCs from individuals with malaria, HIV, and autoimmune diseases exhibit similar transcriptional profiles, suggesting a potentially shared differentiation trajectory across a variety of immune contexts [264]. While T-bet was believed to be essential for ABC development [248, 251, 265], recent findings indicate that T-bet is not strictly needed, as CD11c⁺ ABCs can still arise in infection and autoimmune models even in the absence of this transcription factor [266, 267]. In addition, Zeb2 has recently been identified as a key transcription factor involved in ABC development both in mice and humans [268, 269], suggesting that it may play a role in imposing a shared transcriptional program of ABCs across different contexts.

Mechanistically, ABCs are thought to arise following BCR stimulation in an inflammatory cytokine milieu, with their differentiation requiring additional signals from endosomal TLRs, as well as from IFN-γ and IL-21 [242, 249, 266, 270–276]. Growing evidence strongly supports the view that ABCs are not merely byproducts of inflammation but also, in most cases, antigen-experienced cells. It has been suggested that in autoimmune settings, ABCs originate from B cells that have encountered self-antigen [255, 269, 274, 275, 277]. In support of this, a recent study has shown that anergic B cells, which emerge following chronic self-antigen engagement, can be converted into ABCs and are more prone than naïve B cells to acquire an ABC phenotype under in vitro conditions that promote ABC differentiation [278]. Furthermore, ABCs have been found within the antigen-specific B cell pool following immunization in both mice and humans [221, 268, 279]. Some ABCs can express classical MBC markers [221, 255, 277, 280], carry somatically hypermutated BCRs [255, 277, 281], and respond to antigen restimulation [221, 280]. Moreover, ABC frequencies decrease significantly in patients with mutations affecting CD40/CD40L [276], and ABCs do not develop in mice with fixed BCR specificity under steady-state conditions [278], further supporting the notion that antigen encounter and, at least in some cases, T cell help is required for their formation.

Despite extensive investigations in recent years, many questions regarding ABC differentiation, homeostasis, and functions remain to be answered. For example, the extent to which the ABC compartment is maintained by self-renewal versus replenishment by newly activated B cells remains unclear. Moreover, the relative contributions of GC-dependent and GC-independent pathways to the ABC pool remain unclear. A recent study using GC-specific fate mapping demonstrated that, following acute viral infection in mice, the majority of antigen-specific ABCs are generated via a GC-independent pathway [221]. However, whether this holds true across other immune contexts that give rise to ABCs remains to be elucidated.

The functions of ABCs across different immunological contexts remain an active area of investigation. A recent study demonstrated that a reduction in ABC numbers through B cell-specific deletion of Zeb2 significantly improved disease outcomes in a lupus mouse model [269]. Similarly, depletion of CD11c⁺ or T-bet⁺ B cells decreased autoantibody levels and ameliorated disease in other autoimmune models [254, 255, 274]. While the latter depletion strategy may affect other activated B cell subsets, the findings suggest that ABCs can play an active, pathogenic role in autoimmunity. In the context of infection, B cell-specific deletion of Zeb2 resulted in decreased numbers of GCBCs during persistent *Plasmodium* infection, indicating a potentially beneficial role for ABCs in sustaining humoral immune responses [268]. Taken together, these findings support the idea that ABCs are functionally involved in shaping humoral immune responses, contributing to protective immunity during infection and sustaining autoreactive responses in autoimmunity. These observations reinforce the notion that ABCs are not merely byproducts of chronic immune activation but represent active players in B cell-mediated immunity. It remains to be investigated whether these cells play a role in autoimmunity and the response to pathogens predominantly through differentiation to antibody-producing PCs, antigen presentation to T cells, or both.

The precise function of ABCs upon reactivation remains incompletely understood. Early in vitro studies indicated that ABCs exhibit limited PC differentiation capacity [245, 246], raising questions about their effector potential. However, later work suggested that the lower PC differentiation observed in earlier studies may have been influenced by the mode of stimulation. Indeed, in contrast to soluble anti-Ig antibodies, membrane-bound anti-Ig effectively drove PC differentiation from ABCs, and it was suggested that soluble antibodies may dampen BCR signaling in these cells through inhibitory receptors such as FcRL5 and FcγRIIB [282]. Moreover, some studies suggest that ABCs are in fact transcriptionally and functionally primed for PC differentiation. This notion is supported by the expression of PC-associated genes by ABCs [221] and the increased propensity of these cells to differentiate into PCs in T cell coculture experiments [272, 283]. Given that ABCs are mostly studied in models involving chronic BCR stimulation, such a propensity would fit with the idea that they may be epigenetically programmed by sustained antigen engagement to favor terminal differentiation into PCs, similar to what has been proposed for conventional MBCs [139]. Nevertheless, a recent study involving adoptive transfer of ABCs between mice of the same autoimmune strain has shown that in addition to PC differentiation, ABCs are capable of both differentiating into GCBCs and self-renewing [255]. Whether this apparent functional plasticity is shaped by the degree of antigenic stimulation experienced by different ABCs prior to restimulation remains unclear. Overall, although the development and function of ABCs remain to be fully elucidated, gaining further insight into their behavior may help clarify how MBCs can develop, persist and function under conditions of chronic antigen exposure.

## Concluding remarks and open questions

In this review, we discuss advances in our understanding of MBC development and how key parameters, such as developmental pathways, tissue localization, and the timing of generation, influence MBC properties. Despite substantial recent progress, many questions concerning MBC differentiation, maintenance, and function remain to be answered.

Until recently, most studies on B cell memory have focused on circulating GC-derived MBCs. Therefore, our understanding of the biology of other MBC subsets, such as tissue-resident MBCs (especially those outside of the lungs) and non-GC-derived _E_MBCs, remains limited. For example, although it is now clear that _E_MBCs can make a very significant contribution to the MBC pool after immunization [92], their involvement in response to chronic infections, the microbiota or participation in autoimmune diseases remains uninvestigated. Moreover, the lack of suitable tools largely precludes investigation of the participation of these cells in secondary immune responses, as existing fate-mapping approaches cannot simultaneously discriminate the progeny of naïve B cells, _E_MBCs, and _GC_MBCs in recall responses. The development of such tools would enable functional characterization of this understudied MBC population.

A hallmark of immunological memory is longevity. While the lifespan of MBCs can range from weeks to decades [230, 284, 285], the determinants underlying this remarkable variability remain unknown and arguably represent one of the most significant unresolved questions in the biology of B cell memory. Many of the parameters discussed in this review, such as developmental origin, activation history, and localization within specific tissue niches, may contribute to the long-term maintenance of MBCs, but this remains to be systematically investigated.
